# Disseminating Self-Help: Positive Psychology Exercises in an Online Trial

**DOI:** 10.2196/jmir.1850

**Published:** 2012-06-25

**Authors:** Stephen M Schueller, Acacia C Parks

**Affiliations:** ^1^University of PennsylvaniaDepartment of PsychologyPositive Psychology CenterPhiladelphia, PAUnited States; ^2^University of California, San Francisco/San Francisco General HospitalDepartment of PsychiatrySan Francisco, CAUnited States; ^3^Hiram CollegeDepartment of PsychologyHiram, OHUnited States

**Keywords:** Internet intervention, positive psychology, self-help

## Abstract

**Background:**

The recent growth of positive psychology has led to a proliferation in exercises to increase positive thoughts, behaviors, and emotions. Preliminary evidence suggests that these exercises hold promise as an approach for reducing depressive symptoms. These exercises are typically researched in isolation as single exercises. The current study examined the acceptability of several multi-exercise packages using online dissemination.

**Objective:**

The purpose of this study was to investigate methods of dissemination that could increase the acceptability and effectiveness of positive psychology exercises. To achieve this goal, we compared the use of positive psychology exercises when delivered in packages of 2, 4, or 6 exercises.

**Methods:**

Self-help–seeking participants enrolled in this study by visiting an online research portal. Consenting participants were randomly assigned to receive 2, 4, or 6 positive psychology exercises (or assessments only) over a 6-week period. These exercises drew from the content of group positive psychotherapy. Participants visited an automated website that distributed exercise instructions, provided email reminders, and contained the baseline and follow-up assessments. Following each exercise, participants rated their enjoyment of the exercise, answered how often they had used each technique, and completed outcome measures.

**Results:**

In total, 1364 individuals consented to participate. Attrition rates across the 2-, 4-, and 6-exercise conditions were similar at 55.5% (181/326), 55.8% (203/364), and 52.7% (168/319) respectively but were significantly greater than the attrition rate of 42.5% (151/355) for the control condition (χ^2^_3 _= 16.40, *P *< .001). Participants in the 6-exercise condition were significant more likely than participants in the 4-exercise condition to use both the third (*F*_1,312 _= 5.61, *P *= .02) and fourth (*F*_1,313 _= 6.03, *P *= .02) exercises. For 5 of the 6 exercises, enjoyment was related to continued use of the exercise at 6-week follow-up (*r*’s = .12 to .39). All conditions produced significant reductions in depressive symptoms (*F*_1,656 _= 94.71, *P *< .001); however, a significant condition by time interaction (*F*_3,656 _= 4.77, *P *= .003) indicated that this reduction was larger in the groups that received 2 or 4 exercises compared with the 6-exercise or control condition.

**Conclusion:**

Increasing the number of exercises presented to participants increased the use of the techniques and did not increase dropout. Participants may be more likely to use these skills when presented with a variety of options. Increasing the number of exercises delivered to participants produced a curvilinear relationship with those in the 2- and 4-exercise conditions reporting larger decreases in depressive symptoms than participants in the 6-exercise or control conditions. Although research generally offers a single exercise to test isolate effects, this study supports that studying variability in dissemination can produce important findings.

## Introduction

Depression is a major global public health issue affecting approximately 121 million people worldwide, a higher prevalence than any other mental disorder [1]. Costing an estimated US $43 billion per year in treatment, depression is the leading cause of disability and lost productivity worldwide [2]. Furthermore, depression is a chronic disease; once an individual has experienced his or her first episode, he or she is 2 to 3 times more likely to experience subsequent episodes [3]. The extent of the depression epidemic is furthered by an additional proportion of the population reporting depressive symptoms that do not meet criteria for a major depressive episode. Mild to moderate depressive symptoms are at least as prevalent as major depressive disorder, and many individuals with subthreshold depressive symptoms experience substantial functional impairment [4,5]. Furthermore, subthreshold symptoms are a strong risk factor for developing major depression in the future [6].

A growing awareness of the burden caused by major depression and impairment due to subclinical symptoms has fostered interest in strategies that promote mental health in individuals before they have experienced full episodes of major depression. One promotion strategy, highlighted by positive psychology, is to improve mental health by increasing the frequencies of positive emotions, behaviors, and cognitions rather than targeting negative emotions and thoughts. Furthermore, the burden of depression might also be reduced through developing nontraditional methods of disseminating mental health treatments to increase access to these treatments. The Internet is one such method to help deliver empirically validated treatments to a broader audience. This study combines these strategies and nontraditional methods of disseminationinto a single investigation of positive psychology techniques disseminated through an Internet-based system.

### The Potential of Positive Psychology

Positive psychology focuses on factors that help promote optimal well-being and investigates the functional consequences of these states [7]. A recent theory by Seligman proposes five pathways to well-being: positive emotions, engagement, relationships, meaning, and achievement [8]. Seligman posits that these pathways are unique predictors of well-being, each of which people pursue and value in their own right.

However, these pathways are linked not only to higher levels of self-reported psychological well-being but also to tangible functional benefits. Experimental and longitudinal studies have found that increased positive emotions lead to social, occupational, and health benefits [9]. Gratitude, for example, promotes both the formation and the upkeep of friendships [10]. Correlational evidence links higher reported focus on engaging and meaningful activities to greater occupational success and higher incomes [11]. Consistent findings suggest that stronger social relationships reduce mortality and help us cope with life stressors [12].

Furthermore, psychological well-being in general, and positive emotion in particular, appear to promote meaning-making in stressful situations, which in turn leads to more effective coping [13]. In a study examining the role of positive emotions in psychological resilience, individuals who experienced positive emotions in the weeks immediately following September 11th were less likely to experience increased depressive symptom levels, controlling for baseline symptoms [14]. Even more interestingly, a recent study showed that increased psychological well-being is a significant protective factor from the occurrence of future episodes of major depression [15]. Indeed, this effect remained even when controlling for baseline levels of depressive symptoms, suggesting that positive mental health is different from merely the absence of psychological distress [16]. Thus, increasing well-being might not only increase positive factors but also reduce negative states and psychopathology.

### Positive Psychology Interventions

A variety of researchers and practitioners have translated theories and findings based on positive emotions, well-being, and interpersonal functioning (ie, gratitude, kindness, positive responding, and savoring) into cognitive and behavioral practices deemed positive interventions. The most common outcome measures used to evaluate these interventions reflect aspects of subjective well-being and include increasing cognitive evaluations (such as life satisfaction or happiness), promoting positive emotions, and decreasing negative emotions (or reducing depressive symptoms).

In one of the first empirical investigations of an intervention aimed to increase happiness, Fordyce provided college students with a course that taught participants to mimic the behaviors of happy people [17]. These skills included behavioral recommendations such as increasing physical and social activity and cognitive strategies such as reducing one’s expectations and cultivating a present-focused orientation. This course led to significant improvements in happiness compared with a placebo control condition. More recent research has focused on isolating individual factors and delivering single interventions. A selection of individual exercises with various targets (gratitude, strengths, and reminiscence) are reviewed below.

#### Gratitude

Gratitude is an emotion related to the reflection on something good that has happened and an acknowledgment of who or what was responsible. As previously mentioned, higher levels of gratitude are linked to improved relationships and coping with stressors [10,14]. One exercise designed to increase gratitude asks participants to reflect each day on three good things that happened by writing these things down in a journal [18]. College students who noted good things reported increased levels of positive affect, increased levels of exercise, better sleep quality, and fewer physical symptoms compared with a control condition where participants wrote about daily hassles. A further investigation of gratitude journaling found that participants benefited more when completing longer weekly entries as opposed to shorter more frequent entries [19]. These results suggest that although even brief techniques can benefit individuals, the strategies must push participants to make large enough changes to be different than their typical routine.

One study comparing five different positive psychology exercises head-to-head used two different gratitude-boosting strategies [20]. One was a replication of gratitude journaling that also asked participants to reflect on the cause of the positive event, and the other required participants to write a gratitude letter to someone that they never had a proper chance to thank and to schedule a “gratitude visit” to read the letter to the respondent. Participants completed each exercise for one week and were followed for six months afterwards. The gratitude visit produced the largest initial gains in happiness; however, at three months, these participants were no happier than they had been at baseline. The gratitude journaling or the three good things exercise did not display benefits for participants until one month after the intervention, but these participants maintained these gains at 3- and 6-month follow-ups.

#### Strengths

In addition to examining the benefits of gratitude, the previous study also examined the benefits of encouraging participants to use one of their “signature strengths.” A signature strength is based on a classification of positive characteristics of individuals that are morally valued in general, that are fulfilling, and that are trait-like [21]. In this exercise, participants complete an evaluation of their strengths that provides them with feedback as to the ranking of their individual strength profile that highlights their top five signature strenghts. Participants who practiced using their signature strengths each day showed increase happiness and decreased depressive symptoms compared with participants in a placebo control group that wrote about early memories.

#### Reminiscence

Similar to gratitude journaling, which encourages participants to identify and note good things during each day, looking back on one’s life and reflecting on positive moments is another strategy to promote well-being. Reminiscence interventions lead to a variety of emotional benefits, especially among elderly individuals. People who tended to reflect on the positive moments of their life reported increased ability to savor life and had higher levels of positive emotions [22]. A program that encouraged people to set aside 10 minutes twice a day for one week to reflect on positive moments in their life showed that this led to improved mood compared with individuals who thought about current interests or concerns [22].

### The Efficacy of Positive Interventions

Thus, various strategies can help promote individual well-being. A recent meta-analysis demonstrated that compared with control conditions, positive interventions lead to reliable and moderate boosts in well-being and improvements in depressive symptoms [23]. Although many of the interventions were brief, single intervention techniques, moderation analysis revealed higher effect sizes associated with “shotgun” approaches, that is, packages that incorporate multiple positive psychology strategies. In light of this, the way to produce the most efficacious intervention might be to combine techniques from previously validated individual exercises into an intervention package.

Positive psychotherapy (PPT) adopted this approach by drawing content from single exercises to create individual and group formats delivered by a single clinician [24]. PPT is a novel and distinct therapy technique that focuses on positive emotions, engagement, and meaning rather than targeting negative states and depressive symptoms. Individual PPT was piloted in a 14-session model for patients with a major depressive disorder recruited from a university counseling center. In a 12-week trial, individual PPT compared with eclectic counseling or eclectic counseling plus medication led to greater improvements in symptoms of depression at the end of treatment [24]. Group PPT was tested in a 6-week, 6-session model for groups of 8 to 11 mild-to-moderately depressed university students. Compared with students assigned to an assessment-only control group, students assigned to group PPT experienced significantly greater symptom reduction and improvements in life satisfaction, and these improvements remained at the 1-year follow-up [24]. These studies indicate the feasibility of these techniques to reduce depressive symptoms in individuals with clinical and subclinical levels of depression. The design of the investigation that we report in this paper translated the individual exercises used in group PPT for online dissemination.

Other programs that have combined content from individual exercises into longer and more involved intervention packages have been delivered in a variety of settings including schools and have also been delivered to military personnel [25,26]. Although outcome studies of these programs are still pending, the reaction from participants has been extremely positive, illustrating the widespread appeal of these techniques. Disseminating these programs, however, is limited by the need to train personnel and the ability of the programs to reach others. A meta-analysis of the school-based Penn Resiliency Program found that only trials delivered by highly trained research team providers produced significant gains [27]. Improving the dissemination of these techniques should be a focus of future research. The study we report here was intended to contribute to this goal.

### Taking Positive Psychology Online

The brief duration and universal appeal of these strategies make positive psychology interventions ideal candidates for online administration. In fact, one of the first empirical tests of positive psychology interventions was conducted via the Internet using a convenience sample drawn from visitors to the website linked to Seligman’s book *Authentic Happiness *[28]. The Internet allows dissemination of these techniques throughout the world, anywhere participants have Web access, rather than having to rely on trained professionals to provide access to these techniques. Positive psychology techniques also lend themselves to Internet research, as these techniques might be less stigmatizing than other interventions that deal with difficulties and deficits. In positive psychology exercises, participants talk about the best aspects of themselves, reflect on what is going well in their lives, and reach out to others in their social network in appreciation. The three good things exercise was even integrated into Facebook through an application that provided users with tools to complete the gratitude journaling online and share with friends in their network [29]. Although the application was not evaluated for efficacy, availability through a Facebook application did lead to increased usage. People using the application on Facebook were roughly twice as likely to report good things compared with users of a three good things Facebook *group*, which is distinct from an application in that it is merely a place where people can post and has no features [29].

Researchers have also used other platforms to promote dissemination of positive psychology interventions. Several individual exercises have been incorporated into *Live Happy*, a positive psychology app developed for the iPhone. In a naturalistic evaluation, participants who used the app reported increases in their mood and happiness throughout a 2-week period [30]. Participants who completed more activities within the app reported larger boosts in well-being than those who used the app less frequently.

Positive psychology strategies have also been used to augment other interventions to increase usage. In a study of an online body dissatisfaction intervention, participants who completed a gratitude diary were twice as likely to complete the intervention compared with a thought-monitoring and cognitive-restructuring group [31]. This relates to the theory underlying PPT, which contends that focusing on strengths and positive emotions fosters engagement and meaning more so than other techniques. Positive psychology techniques might be more enjoyable and acceptable than other interventions. This is consistent with the overwhelming positive feedback from military and school-based positive psychology programs.

### Current Study

Thus far, we have discussed existing positive psychology approaches and two important limitations of these approaches: feasibility (most interventions are administered in person, often by a therapist) and length (most interventions consist of a single exercise). The goal of the current study was to address both of these issues by offering a Web-based intervention that would last between 2 and 6 weeks. We aimed to systematically examine the effects of increased intervention length on adherence and efficacy, while also gaining basic usability data about the acceptability of an online positive psychology intervention that offers more than a single exercise.

The current study adapted group PPT into an online environment in order to investigate these aims. As previously stated, a meta-analysis of existing positive interventions demonstrated that longer interventions and packages with several techniques typically reported larger effect sizes. Our Web-based trial of positive interventions was designed to investigate these questions by comparing sets of 2, 4, or 6 exercises as well as an assessment-only control condition in terms of usage of the techniques (attrition and self-report) and efficacy (decreases in depressive symptoms). Exercises were drawn from group PPT and included active-constructive responding, a gratitude visit, life summary, three good things, savoring, and strengths. Table 1 provides brief descriptions of each of the exercises.

**Table 1:** Descriptions of individual exercises

Name of Exercise	Description of Exercise	Empirical Support (authors, publication year)
Active-constructive responding	Participants learn to respond positively to good news shared by others by lengthening the conversation and helping the sharer relive the experience.	Gable et al, 2004 [32]
Gratitude visit	Participants write a letter of gratitude and read it aloud to the target of the letter.	Seligman et al, 2005 [20]; Seligman et al, 2006 [24]
Life summary	Participants write a summary of how they would want their life expressed to their progeny.	Seligman et al, 2005 [20]; Seligman et al, 2006 [24]
Three good things	Participants identify three things that went well each day and why. These good things are kept in a gratitude journal throughout the week.	Emmons and McCullough, 2003 [18]; Seligman et al, 2005 [20]
Savoring	Participants are instructed to take time to focus intently on a positive experience 2 to 3 times each day.	Seligman et al, 2006 [24]
Strengths	Participants take the Values in Action Strengths Questionnaire and receive individualized feedback about their strengths and are instructed to use one of their top five (signature) strengths each day.	Seligman et al, 2005 [20]; Seligman et al, 2006 [24]

We posited that similar to the in-person group PPT, the online administration would lead to significantly greater decreases in depressive symptoms. Furthermore, consistent with the findings of the meta-analysis of positive interventions, we predicted that participants receiving more exercises would experience greater benefits.

## Methods

### Recruitment

Participants were recruited through a research portal associated with the University of Pennsylvania’s Positive Psychology Center and Seligman’s book *Authentic Happiness *[28]. A link available on the websites www.authentichappiness.org and www.ppresearch.sas.upenn.edu invited interested individuals to participate in a research study on positive psychology exercises.

### Study Procedures 

These landing sites offered participants a variety of assessment and research opportunities. From the initial website, individuals could access and receive feedback on questionnaires assessing emotions, engagement, meaning, and life satisfaction. Those who clicked on the link for a research study on positive psychology exercises were informed that they would be randomly assigned to conditions receiving either 2, 4, or 6 positive psychology exercises or an assessment-only control condition over a 6-week period and would be asked to return to the website each week to complete assessment measures. Participants completed consent online by reading the informed consent document and providing an electronic signature.

After completing the baseline questionnaires that assessed demographics as well as mood and depressive symptoms, participants were randomly assigned to one of the four groups. Those assigned to one of the three treatment conditions received instructions for the first positive psychology exercise and were asked to practice that exercise over the course of the following week. Exercises were provided in the following fixed order: three good things, strengths, gratitude visit, savoring, active-constructive responding, and life summary. Participants in the 2-exercise condition, for example, received the first two exercises in this sequence (three good things and strengths) and were encouraged to use these exercises in the following weeks and return to the website to complete follow-up measures. Participants in the assessment-only control condition simply received a message to return the following week to complete additional assessments. Participants in all groups received email reminders each week to return to the site and complete the weekly assessments. After completion of these measures, participants received the next exercise in the sequence if allowed in their study condition.

### Measures

At baseline, participants completed thorough demographics that included age, gender, ethnicity, marital status, educational attainment, income, and region (first three digits of their zip code). Participants also responded to questions regarding history of psychiatric illness and treatment. During the baseline assessment and at each weekly follow-up, participants also completed the Center for Epidemiological Studies-Depression scale (CES-D), a 20-item self-report scale designed to measure the current level of depressive symptoms [33]. The CES-D contains items assessing negative symptoms such as depressed mood (eg, “I felt depressed”) and appetite disturbance (eg, “I did not feel like eating; my appetite was poor”) as well as positive symptoms (eg, “I felt hopeful about the future”) which are reverse-coded to create a single summary scale of depressive symptoms.

### Data Analyses 

In order to investigate the hypotheses of the current study, we conducted chi-square tests comparing attrition rates across the four conditions. Given the high rates of attrition in Internet-based studies, we conducted analyses of the *completers*, that is, participants who completed the pretest and posttest measures. We used repeated measures analysis of covariance of the completers to assess changes over the 6-week intervention period in depressive symptoms across the 4 conditions while controlling for baseline levels of symptoms.

## Results

### Sample

Participants included all those who visited the website and consented to participate during a 20-month period from February 2007 through November 2008. Participants were predominantly women (1038/1364, 76.1%) and middle aged with a mean age of 42.38 years (SD 12.08). The largest proportion of participants lived in the United States (743/1364, 54.5%), although many participants were from other countries (621/1364, 45.5%). Most participants were married (602/1364, 44.1%), while 35.0% (477/1364) reported never having been married, and the remaining 20.9% (285/1364) were separated, divorced, or widowed. This was a highly educated sample with 77.2% (1053/1364) of participants reporting they had received a bachelor’s degree or higher. Income was generally high as 24.7% (337/1364) reported earning over US $99,000, and only 11.7% (160/1364) reported earning under US $20,000.

### Attrition

**Figure 1:**
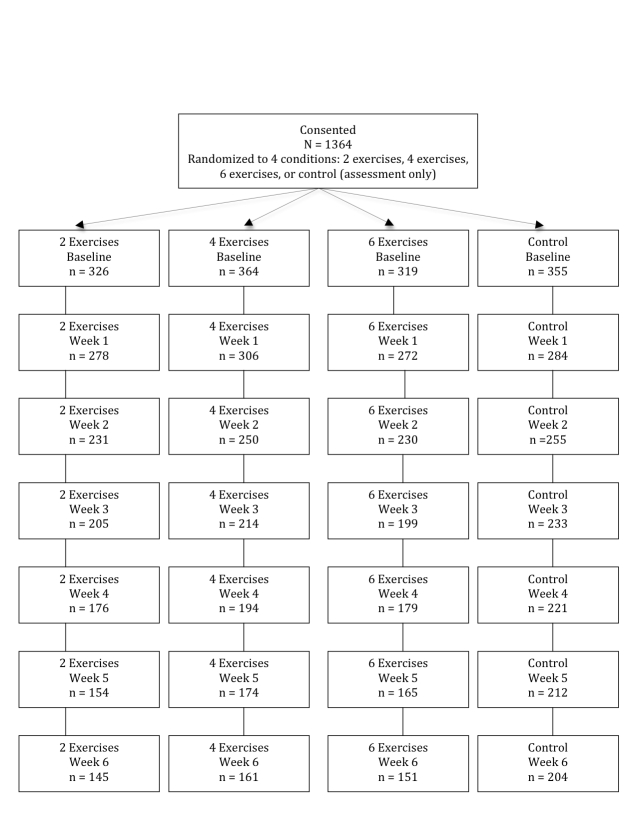
Flow of participants through the trial.

Figure 1 displays the consolidated standards of reporting trials (CONSORT) diagram for the study. Of the 1364 participants who consented to participate, 1140 (83.6%), 966 (70.8%), 851 (62.4%), 770 (56.4%), 705 (51.7%), and 661 (48.4%) completed measures each week at the week 1 through week 6 assessments respectively. At the end of the 6-week assessment period, attrition rates across the active treatment conditions were similar, that is, 55.5% (181/326) for 2 exercises, 55.8% (203/364) for 4 exercises, and 52.7% (168/319) for 6 exercises, but all were significantly greater than the 42.5% (151/355) attrition rate for the control condition (χ^2^_3 _= 16.40, *P *< .001). 

### Use of Exercises

Compliance (ie, exercise usage) was assessed for each exercise at the 1-week assessment following its assignment and again at each of the subsequent assessments. There were no significant differences between the 6 exercises on use of the techniques. Individuals in the 6-exercise condition were significant more likely than participants in the 4-exercise condition to use both the third (*F*_1,312 _= 5.61, *P *= .02) and fourth (*F*_1,313 _= 6.03, *P *= .02) exercises. At the end of the 6 weeks, the three active conditions differed significantly on the number of days that the techniques were used (*F*_2,358 _= 33.84, *P *< .001). For the 2-exercise condition, mean usage was 6.1 days (SD 3.56); for the 4-exercise condition, mean usage was 7.94 days (SD 4.77); and for the 6-exercise condition, mean usage was 12.1 days (SD 7.36). For 5 of the 6 exercises (all of the 6 with exception of the life summary), enjoyment was related to continued use of the exercise at the 6-week follow-up (*r*’s = .12 to .39, *P*’s < .05).

### Efficacy of Exercises

**Figure 2:**
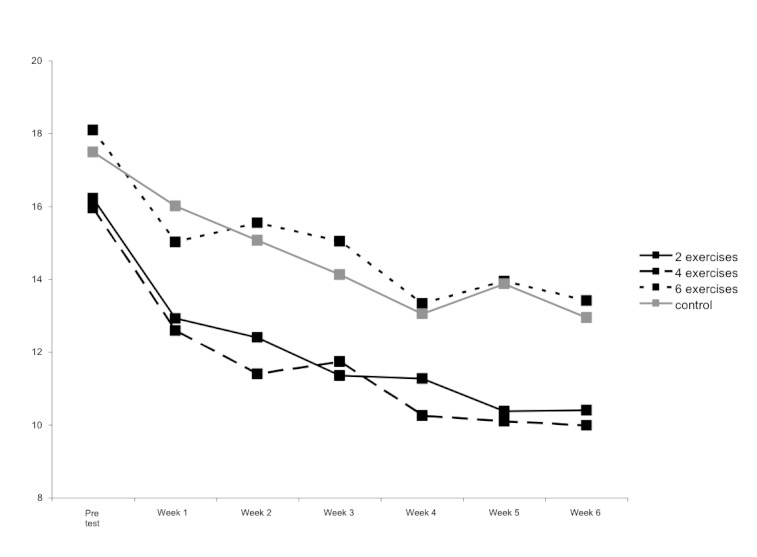
Mean CES-D scores by condition.

To analyze efficacy of the package of interventions, a repeated measures analysis of covariance was conducted on measures of depressive symptoms. At baseline, the average scores on the CES-D were consistent with mild-to-moderate levels of depressive symptoms (mean 16.93, SD 12.64). There were no significant differences between the groups on baseline levels of depressive symptoms: means were 16.22, 15.95, and 18.10 for participants in the 2-, 4-, and 6-exercise conditions respectively, while the mean for those in the control condition was 17.49. A significant main effect of time (*F*_1,656 _= 94.71, *P *< .001) demonstrates that depression scores dropped over time in all conditions. Furthermore, a significant condition by time interaction (*F*_3,656 _= 4.77, *P *= .003) indicates that these decreases differed across the conditions. Post-hoc Tukey HSD (honestly significant difference) tests analyzed the differences among the four conditions. These analyses showed that at the end of the intervention period, participants in both the 2-exercise condition and the 4-exercise condition reported significantly fewer depressive symptoms than those in the 6-exercise condition and numerically (but not significantly) lower than participants in the control condition. Depressive symptoms reported by participants in the 6-exercise condition did not differ significantly from those reported by participants in the control condition. Figure 2 displays mean CES-D scores by condition at each weekly assessment. Estimated marginal means in the CES-D were 12.31, 11.95, 15.89, and 14.53 for the 2-, 4-, and 6-exercise groups and control condition, respectively.

We also analyzed whether use of the exercises played a mediating role in the changes in depressive symptoms. We conducted regressions predicting changes in depressive symptoms while controlling for baseline symptoms investigating both linear and curvilinear relationships with the number of days that exercises were practiced. Neither term was significant, suggesting that the number of days of engagement did not relate to changes in depressive symptoms.

## Discussion

The results of this study highlighted considerations regarding the implications of providing additional content to participants in Internet-based interventions. First, increasing the amount and variety of content did not lead to differences in attrition rates. Participants in the 2-, 4-, and 6-exercise conditions dropped out at roughly the same rate. Second, receiving more exercises corresponded to higher use of these exercises. Participants who received 6 exercises in total reported greater use of the gratitude visit and savoring exercises than those participants who only received 4 exercises. Furthermore, participants in the 6-exercise condition reported increased use summing across all the techniques at follow-up. Participants in the 6-exercise condition did report decreases in depressive symptoms after completing the interventions; however, they did not differ in reduction of depressive symptoms compared with those in the control group. Participants who received 2 or 4 exercises, on the other hand, both experienced significant reductions compared with participants in the control group and those in the 6-exercise condition. This suggests a curvilinear relationship such that some exercises are better than none but that 6 exercises was associated with fewer gains.

The main aim of this study was to translate a previously validated package of positive psychology exercises for online dissemination. Indeed, in this study, participants who, on average, experienced mild-to-moderate depressive symptoms at baseline did experience reductions in depressive symptoms. A further aim of this study was to evaluate a dose-response relationship to determine if receiving more elements of the intervention led to differences in adherence or efficacy. Increasing the content delivered to participants did not reduce rates of follow-up and corresponded to increases in reported rates of usage of the strategies taught through the intervention. This result might be because increasing the number of choices given to someone will increase the likelihood that he or she will find an exercise (or several exercises) that is a good fit and thus continue to use the technique.

Despite other research that has demonstrated a relationship between adherence to the intervention and efficacy [34,35], this study found that participants who received less content actually made larger gains than participants who received more content. This did not support our hypothesis that providing more exercises would lead to increased benefits. Instead, the results demonstrate a more complex relationship between the amount of content delivered and efficacy of the set of techniques. It might be that increasing the diversity of exercises leads to participants splitting their time among the techniques and not focusing on any of the techniques long enough to benefit substantially.

This finding, if replicated, provides the most constructive advice for those interested in creating and disseminating Internet-based interventions: do not overwhelm the participants. Past research in positive psychology interventions has found that variety is an important moderator of intervention gains and that several techniques are more effective than a single technique [23,36]. In one such study, participants who were allowed to vary acts of kindness from a previously specified list over a 10-week period received greater gains in happiness compared with participants who were required to repeat the same acts of kindness each week [36]. Consistent with the results of the current study, however, participants who were allowed complete freedom to create new acts each week actually did worse than those who varied the acts but used the previously generated list.

These findings taken together indicate that increasing content might provide additional skills and tools to participants up to a point. But after this point, participants might not know which tools to use when or might become overwhelmed by the number of choices afforded to them. Indeed, the vast literature on choice suggests that although multiple options are beneficial, the relationship between choice and human happiness is curvilinear with too many options actually leading to various psychological and economic factors (time trade-offs, remorse, rumination, etc) that decrease psychological well-being [37]. Although the issue of depth versus breath of options is still an open and important empirical consideration, it appears that in some cases at least less is more.

The findings of this study are tempered by its limitations. Similar to other Internet-based studies, participants in this study were overwhelming female, highly affluent, and well educated. Although this study was an open trial and participants throughout the world could (and did) visit the website, the sample was not representative of the world population. In part, this might be due to the recruitment procedure, which advertised via a website associated with a popular positive psychology book and the University of Pennsylvania’s Positive Psychology Center. Future research on these interventions should adopt alternative recruitment procedures to draw people into the study rather than relying on participant flow from existing resources. Another limitation is that although this study did experimentally manipulate the amount of content participants were offered, the content was presented through an automated system, and thus we cannot be sure whether participants actually read and understood the instructions. Furthermore, instructions were delivered over subsequent weeks, and participants were asked to use one activity per week although in the real world, participants are more likely to receive several potential activities at once and use them at will. It would be worthwhile to further investigate the question of depth versus breath of interventions in a format where participants can access the additional content simultaneously.

Overall, this study provides a further illustration of the flexibility and adaptability of positive psychology interventions. As a majority of the existing research on Internet-based interventions has evaluated cognitive-behavioral based techniques, future work should continue to integrate other validated strategies to promote psychological well-being. The science of Internet interventions can be advanced through expanding options and strategies to promote worldwide well-being. 

## Abbreviations

CES-D: Center for Epidemiological Studies–Depression scale
CONSORT: consolidated standards of reporting trials
HSD: honestly significant difference
PPT: positive psychotherapy
